# Reaching Minimal Clinically Important Difference, Substantial Clinical Benefit, and Patient-Acceptable Symptomatic State for Patient-Reported Outcome Measures following Arthroscopic Rotator Cuff Repair Does Not Correlate with Patient Satisfaction

**DOI:** 10.3390/jcm13092550

**Published:** 2024-04-26

**Authors:** Adam Z. Khan, Alayna K. Vaughan, Zachary S. Aman, Mark D. Lazarus, Gerald R. Williams, Surena Namdari

**Affiliations:** 1 Southern California Permanente Medical Group, 13652 Cantara Street, Bldg 2, Panorama City, CA 91402, USA; 2 Rothman Orthopaedics, 925 Chestnut Street, 5^th^ Floor, Philadelphia, PA 19107, USAsurena.namdari@rothmanortho.com (S.N.)

**Keywords:** shoulder, rotator cuff, patient-reported outcomes, patient satisfaction

## Abstract

**Purpose:** Minimal clinically important difference (MCID), substantial clinical benefit (SCB), and patient acceptable symptomatic state (PASS) serve as metrics to gauge orthopedic treatment efficacy based on anchoring questions that do not account for a patient’s satisfaction with their surgical outcome. This study evaluates if reaching MCID, SCB, or PASS values for American Shoulder and Elbow Surgeons score (ASES), Single Alpha Numeric Evaluation (SANE), Simple Shoulder Test (SST), and Visual Analog Score (VAS) for pain following arthroscopic rotator cuff repair (RCR) correlates with overall patient satisfaction. **Methods:** This was a single-institution, retrospective study of patients who underwent RCR from 2015 to 2019. Pre-operative and 2 year postoperative ASES, SANE, SST, and VAS scores were recorded. Patients underwent a survey to assess: (1) what is your overall satisfaction with your surgical outcome? (scale 1 to 10); (2) if you could go back in time, would you undergo this operation again? (yes/no); (3) for the same condition, would you recommend this operation to a friend or family member? (yes/no). Spearman correlation coefficients were run to assess relationship between reaching MCID, SCB, or PASS and satisfaction. **Results:** Ninety-two patients were included. Mean preoperative ASES was 51.1 ± 16.9, SANE was 43.3 ± 20.9, SST was 5.4 ± 2.9, and VAS was 4.6 ± 2.1. Mean 2 year ASES was 83.9 ± 18.5, SANE was 81.7 ± 27.0, SST was 9.8 ± 3.2, and VAS was 1.4 ± 1.9. Mean patient satisfaction was 9.0 ± 1.9; 89 (96.7%) patients would undergo surgery again and recommend surgery. Correlation for reaching PASS for SANE and satisfaction was moderate. Correlation coefficients were very weak for all other outcome metrics. **Conclusions:** Reaching MCID, SCB, and PASS in ASES, SANE, SST, or VAS following RCR did not correlate with a patient’s overall satisfaction or willingness to undergo surgery again or recommend surgery. Further investigation into the statistical credibility and overall clinical value of MCID, SCB, and PASS is necessary.

## 1. Introduction

Establishing objective outcome measures and patient benchmarks following orthopedic procedures is paramount to setting patient expectations preoperatively and determining long-term surgical success. Minimal clinically important difference (MCID), Substantial Clinical Benefit (SCB), and Patient Acceptable Symptomatic State (PASS) are three recently established benchmark values that are being set for patient-reported outcomes in the orthopedic literature throughout various subspecialties [1,2,3,4,5,6,7]. The goal of these outcome instruments is to evaluate treatment effectiveness and establish a metric that is more specific to an individual patient’s outcome.

Following arthroscopic rotator cuff repair, commonly assessed patient-reported outcome measures include the American Shoulder and Elbow Surgeons (ASES) score, the Simple Shoulder Test (SST), the Single Assessment Numeric Evaluation (SANE), and the Visual Analog Scales (VAS) for pain. These four patient-reported outcome measures have been well validated for assessment of pain and function for shoulder-specific conditions [8,9,10].

MCID has been established following both operative [1,2] and nonoperative [3,4] management of rotator cuff tears for ASES, SST, SANE, and VAS. For determination of MCID, a four-question anchoring questionnaire was utilized: “Please rate your response to treatment: none or no improvement (A); poor or some improvement but unsatisfactory (B); good satisfactory improvement (C); and excellent ideal outcome (D)”. For determination of SCB, these same four anchoring questions are utilized. However, while MCID is the value required to see clinical change in a patient’s symptom state, SCB is defined as the value required to see clinical improvement in a patient’s symptom state. SCB has been established for ASES, SANE, and VAS following arthroscopic rotator cuff repair [5,6].

PASS has also been established for ASES, SANE, and VAS following arthroscopic rotator cuff repair [5,6]. The anchoring question utilized for PASS is as follows: “Taking into account all the activities you have during your daily life, your level of pain, and also your functional impairment, do you consider that your current state is satisfactory? (Yes/No)”. There is currently no established SCB or PASS metric for SST following arthroscopic rotator cuff repair.

These anchoring questions utilized for MCID, SCB, and PASS do not account for a patient’s overall satisfaction with having undergone surgery, willingness to undergo surgery again if they were given the option, or willingness to recommend surgery to a friend or family member for the same condition. Jones et al. [7] recently published a systematic review of shoulder-related MCID values. This study argued that multiple studies that have reported MCID values for shoulder studies to date have poor study methodology and lack enough data to report statistical credibility. This brings about the questions of how these published MCID values account for true surgical value, postoperative benefit patients experience, or their overall satisfaction with the performed operation.

The purpose of this study was to evaluate the ability of reaching MCID, SCB, and PASS for different shoulder outcomes metrics (ASES, SANE, SST, and VAS) to predict patient satisfaction and willingness to undergo or recommend arthroscopic rotator cuff repair. A minimum follow-up of 2 years was utilized for this study.

## 2. Methods

### 2.1. Patient Demographic Characteristics and Selection

This was a single-institution, retrospective, cohort study of all patients who underwent primary arthroscopic rotator cuff repair from January 2015 to December 2019 by one of nine fellowship-trained shoulder and elbow surgeons. Institutional Review Board study approval was obtained prior to initiation of this investigation (Control #22E.020). Inclusion criteria included patients over the age of 18 who underwent primary arthroscopic rotator cuff repair and had complete preoperative as well as 2-year minimum postoperative ASES, SANE, SST, and VAS scores. Patients undergoing revision surgery or concomitant graft augmentation, superior capsular reconstruction, or tendon transfer were excluded. Patients who had a complication or underwent revision surgery within the 2-year follow-up period causing a deviation from the standard postoperative protocol were also excluded.

### 2.2. Outcome Metrics

Baseline patient characteristics including age and gender were recorded. Patients were followed postoperatively based on the individual surgeon’s postoperative protocol and seen at regular intervals of 2 to 4 weeks, 3 months, and 6 months. Patients were sent online surveys through an automated outcome database for ASES, SANE, SST, and VAS pain scores. This database was queried, and patients with complete preoperative and 2-year outcome scores were contacted and underwent a phone survey in which they were asked one of three questions:

(1) On a scale of 1 to 10, what is your overall satisfaction with your surgical outcome?
(2) If you could go back in time, would you undergo this operation again? (Yes/No)
(3) For the same condition, would you recommend this operation to a friend or family member? (Yes/No)

This survey was conducted by an independent research assistant and not a member of the patient’s care team. This was done to reduce bias in which a patient might falsely inflate their satisfaction if surveyed by their treating surgeon.

Previously established values in the literature for MCID, SCB, and PASS for the four outcome metrics following rotator cuff repair were utilized. For MCID, the following values were utilized: ASES (27.1) [4], SANE (14.9) [8], SST (4.3) [4], and VAS (2.4) [4]. Previously established SCB values were utilized for ASES (26.0) [9], SANE (29.8) [8], and VAS (2.5) [9]. Previously established PASS values were utilized for ASES (86.7) [8], SANE (82.5) [8], and VAS (1.7) [9]. No previously established metric for SST, SCB, or PASS were established in the literature, and, therefore, these values were omitted from the analysis.

### 2.3. Statistical Analysis

Following collection of data, patients were stratified into categories based on reaching MCID, SCB, or PASS in each of the four outcome measures (ASES, SANE, SST, and VAS). For MCID, patients were also stratified into the number of outcome measures in which they reached MCID.

Spearman correlation coefficients were run to assess the relationship between reaching MCID, SCB, and PASS for the three patient satisfaction outcome metrics above. Correlation was graded as follows: less than 0.19 was very weak, 0.20–0.39 was weak, 0.40–0.59 was moderate, 0.60–0.79 was strong, and 0.80–1.00 was very strong.

## 3. Results

### 3.1. Cohort Patient Characteristics

Ninety-two patients were included and completed the outcome metric survey. Mean patient age was 59.8 ± 9.1. There were 61 (66.3%) male patients and 31 (33.7%) female patients. The mean preoperative ASES was 51.1 ± 16.9, SANE was 43.3 ± 20.9, SST was 5.4 ± 2.9, and VAS was 4.6 ± 2.1. At 2 years postoperatively, mean ASES was 83.9 ± 18.5, SANE was 81.7 ± 27.0, SST was 9.8 ± 3.2, and VAS was 1.4 ± 1.9.

At minimum 2-year follow-up, mean patient satisfaction was 9.0 ± 1.9. Eighty-nine (96.7%) patients would undergo surgery again as well as recommend surgery to a friend or family member.

There were 12 patients (13.0%) with reported satisfaction scores below 8 out of 10. At 2-year follow-up, this subset of patients had a mean ASES of 82.0 ± 28.1, SANE was 67.5 ± 19.2, SST was 10 ± 4.18, and VAS was 1.75 ± 1.86.

### 3.2. Minimal Clinically Important Difference

Fifty-nine patients (64.1%) reached MCID for ASES. There were 76 patients (82.6%) who reached MCID for SANE. There were 45 patients (48.9%) who reached MCID for SST. Finally, there were 55 patients (59.8%) who reached MCID for VAS. In total, 27.1% of patients reached MCID in all four outcome metrics, and 3.3% of patients did not reach MCID in any metric [Table 1].

Regarding the subgroup of three patients (3.3%) that did not reach MCID in any of the four outcome metrics, all three patients reported scores of 10 out of 10 satisfaction, would undergo surgery again, and would recommend surgery to a friend or family member. Similarly, all 17 patients (18.5%) who only reached MCID in one outcome metric had satisfaction scores of 9 or 10 out of 10 (mean 9.64) and would undergo surgery again. One of the 17 patients stated that while he would undergo surgery again, he would not recommend surgery to a friend or family member.

Spearman correlation coefficients were very weak for reaching MCID and all three study outcome metrics [Table 2].

### 3.3. Substantial Clinical Benefit

Regarding SCB metrics, 69 patients (75%) reached SCB for ASES. There were 62 patients (67.4%) who reached SCB for SANE. There were 55 (59.8%) patients who reached SCB for VAS [Table 3].

Spearman correlation coefficients were very weak for reaching SCB and all three study outcome metrics [Table 4].

### 3.4. Patient Acceptable Symptomatic State

Regarding PASS metrics, 56 patients (60.9%) reached PASS for ASES. There were 59 patients (64.1%) who reached PASS for SANE. There were 61 (66.3%) patients who reached PASS for VAS [Table 5].

The Spearman correlation coefficient was moderate for the association between reaching PASS and overall patient satisfaction. The Spearman correlation coefficients were very weak for reaching PASS and wanting to undergo surgery again or recommending surgery to a friend or family member [Table 6].

## 4. Discussion

The main finding of this study is that following arthroscopic rotator cuff repair, there is no statistically significant strong correlation between reaching MCID, SCB, or PASS for four different patient-reported outcome metrics (ASES, SANE, SST, and VAS) and patient satisfaction at 2 years postoperatively. Overall, in this cohort of 92 patients, there was a high rate of patient satisfaction postoperatively (mean 9.0 ± 1.9) with the majority of patients (96.7%) interested in undergoing surgery again as well as willing to recommend surgery to a friend or family member. These findings are consistent with prior work showing a high rate of patient satisfaction following rotator cuff repair [11,12]. This is the first study to evaluate the relationship between patient satisfaction and reaching MCID, SCB, or PASS following rotator cuff repair. Interestingly, there was no strong relationship between these outcome metrics and patient satisfaction; further investigation into the etiology behind these findings is indicated.

In this cohort, there were 12 patients (13%) who had satisfaction scores below an 8 out of 10. Of these 12 patients, 5 (41.7%) reached MCID in all four outcome scores, 3 (25%) reached MCID in three outcome scores, and 4 (33.3%) reached MCID in two outcome scores. These patients had comparable final ASES, SST, and VAS scores to the cohort as a whole. Interestingly, there was a decrease in final SANE score in the group with lower satisfaction scores relative to the cohort as a whole (67.5 vs. 81.7). The SANE score is an overall measurement of a patient’s assessment of their shoulder as a percentage of normal (0–100) and has been validated as an accurate and reliable outcome metric following rotator cuff repair [4,13,14]. While the SANE score (also known as Subjective Shoulder Value or SSV) does not evaluate how a surgical intervention affects specific functional activities, it does give insight into the patient’s overall evaluation of their shoulder. In this study, reaching PASS for SANE at 2 years postoperatively was the only outcome metric that showed a moderate correlation with final patient satisfaction [Table 6].

Regarding the subgroup of patients who did not reach MCID in all four outcome scores (3.3%) or only reached MCID in one outcome score (18.5%), there was a high rate of final patient satisfaction in this cohort. All patients reported a score of 9 or 10 out of 10 for satisfaction and stated they would undergo surgery again. Only one patient in this cohort stated he would not recommend surgery to a friend or family member. The findings of this study suggest that there are factors outside of reaching predetermined metrics of improvement in function and pain that play a role in patient satisfaction with their surgical outcome and willingness to undergo rotator cuff surgery or recommend surgery to friends and family members.

As the focus of care shifts to shared decision-making between patient and clinician, patient satisfaction has become a more significant quality assessment metric utilized by clinicians, insurance companies, and hospital systems [15]. Furthermore, patient satisfaction can play a significant role in patient referrals as well as clinician and hospital reimbursement [16,17]. As shown by the findings of this study, satisfaction is a multidimensional outcome metric that does not appear to necessarily correlate with a patient’s functional outcome or pain scores. Furthermore, patient satisfaction is subject to a degree of cognitive dissonance, where a stark difference can exist between a patient’s functional outcome and surgical satisfaction, with other patient-specific factors playing a significant role in their ultimate satisfaction [18,19]. Ruggiero et al. [20] evaluated patient satisfaction following rotator cuff repair at 32.1 months follow-up. Similar to the findings of this study, they found a high rate of overall patient satisfaction (97.1%) and a statistically significant association between SSV and patient satisfaction. They found no association between age, gender, smoking, medical comorbidities, DASH score, or physical therapy regimen and final patient satisfaction. Ventimiglia et al. [21] evaluated preoperative factors associated with patient satisfaction at 2 years following elective shoulder surgery. They found that higher preoperative pain scores (specifically PROMIS Pain Interference), lower annual income (below $70,000), and higher American Society of Anesthesiologists (ASA) score (ASA > 1) were all independent predictors of lower postoperative satisfaction. Interestingly, in the current study, there was a very weak correlation between reaching MCID, SCB, or PASS in VAS pain scores and overall patient satisfaction at final follow-up.

Recent work by McCahon et al. [22] showed that 25% of rotator cuff repair patients received unexpected bills following surgery, and this could be in excess of $1000 in over half of these cases. When interviewing patients, these authors found that this factor significantly lowered patient overall surgical satisfaction. This is an example of one factor that is not accounted for when looking at a patient’s pain levels or functional outcome yet plays a significant role in overall satisfaction. This speaks to the multifactorial nature of patient satisfaction following rotator cuff repair. A systematic review by Kennedy et al. [23] evaluated the psychosocial factors that have an effect on patient-reported outcomes following rotator cuff repair. This review showed that psychosocial factors such as anxiety and depression can influence patient-reported outcomes following rotator cuff repair surgery. No work has specifically looked at the effects of psychosocial factors on patient satisfaction. Given that satisfaction is a multifactorial and subjective measure, a patient’s mental health status and overall perception can play a significant role in their long-term surgical outcome satisfaction. Evaluating patient anxiety and depression scores was outside the scope of this investigation; however, this is an area of potential future investigation.

There are limitations to this study. This was a retrospective analysis of patients who underwent arthroscopic rotator cuff repair in which we excluded patients who did not complete 2 years follow-up outcome metrics. This may have introduced a selection bias to patients who were willing to complete outcome metric surveys at 2 years postoperatively. Furthermore, given the time point of satisfaction data collection, this study is also limited by some potential recall bias [24,25]. These outcome metrics were completed online, whereas phone surveys were utilized for the satisfaction survey. This may have introduced some bias, as patients may be more likely to inflate their satisfaction over the phone rather than an anonymous online survey. To mitigate some of this bias, we had the survey performed by an independent research assistant and not a member of the patient’s care team. Furthermore, to standardize our results, we excluded patients who underwent complications or reoperation in the 2-year postoperative period. This was to give a more consistent timeline and outcome time period of collecting postoperative outcome metrics but may also have introduced some bias as this population of patients would in theory have lower satisfaction levels. This study was performed at a single institution with shoulder and elbow fellowship-trained surgeons, and therefore the results may not be fully generalizable to other practice settings. We did not evaluate preoperative or postoperative imaging and therefore did not take into consideration rotator cuff tear size or healing rates, which are two factors that can play a role in patient satisfaction and outcome measures [26]. Finally, we did not consider patient-specific factors in our analysis such as age, gender, anxiety, depression, medical comorbidities, socioeconomic status, or insurance status, which could all play a role in a patient’s overall satisfaction. However, we do not believe those weaknesses influence our goal of comparing MCID, SCB, and PASS for different shoulder outcomes metrics (ASES, SANE, SST, and VAS) with patient-reported satisfaction and willingness to undergo or recommend arthroscopic rotator cuff repair.

## 5. Conclusions

Reaching MCID, SCB, and PASS in ASES, SANE, SST, or VAS following arthroscopic rotator cuff repair did not correlate with a patient’s overall satisfaction, willingness to undergo surgery again, or willingness to recommend surgery to a friend or family member. Further investigation into the statistical credibility and overall clinical value of currently defined MCID, SCB, and PASS values for arthroscopic rotator cuff repair is necessary.

## Figures and Tables

**Table 1:** (**A**) Number of patients that reached MCID in ASES, SANE, SST, and VAS at 2 years postoperatively. (**B**) Patients stratified by the number of outcome measures in which they reached MCID.

(**A**)		
**Patient-Reported Outcomes**	**Number of Patients (N = 92)**	
	**Reached MCID (%)**	**Did Not Reach MCID (%)**
ASES	59 (64.1%)	33 (35.9%)
SANE	76 (82.6%)	16 (17.4%)
SST	45 (48.9%)	47 (51.1%)
VAS	55 (59.8%)	37 (40.2%)
(**B**)		
**Number of Outcomes Reached MCID**	**Number of Patients (N = 92)**	
0	3 (3.3%)	
1	17 (18.5%)	
2	23 (25.0%)	
3	24 (26.1%)	
4	25 (27.1%)	

**Table 2:** Spearman correlation coefficients for reaching MCID in ASES, SANE, SST, VAS, and all four outcome measures and outcome metrics of patient overall satisfaction, willingness to undergo surgery again, and willingness to recommend surgery to a friend or family member.

**Reached MCID ASES**	**Spearman Correlation Coefficient**	
Patient Overall Satisfaction	−0.044	Very weak
Willing to Undergo Surgery Again	−0.010	Very weak
Willing to Recommend Surgery	−0.010	Very weak
**Reached MCID SANE**	**Spearman Correlation Coefficient**	
Patient Overall Satisfaction	0.009	Very weak
Willing to Undergo Surgery Again	0.077	Very weak
Willing to Recommend Surgery	0.077	Very weak
**Reached MCID SST**	**Spearman Correlation Coefficient**	
Patient Overall Satisfaction	−0.051	Very weak
Willing to Undergo Surgery Again	−0.065	Very weak
Willing to Recommend Surgery	0.057	Very weak
**Reached MCID VAS**	**Spearman Correlation Coefficient**	
Patient Overall Satisfaction	−0.011	Very weak
Willing to Undergo Surgery Again	−0.026	Very weak
Willing to Recommend Surgery	0.099	Very weak
**Reached MCID all metrics**	**Spearman Correlation Coefficient**	
Patient Overall Satisfaction	−0.021	Very weak
Willing to Undergo Surgery Again	−0.008	Very weak
Willing to Recommend Surgery	0.095	Very weak

**Table 3:** Number of patients that reached SCB in ASES, SANE, and VAS at 2 years postoperatively.

Patient-Reported Outcomes	Number of Patients (N = 92)	
	Reached SCB (%)	Did Not Reach SCB (%)
ASES	69 (75%)	23 (25.0%)
SANE	62 (67.4%)	30 (32.6%)
SST	N/A	N/A
VAS	55 (59.8%)	37 (40.2%)

**Table 4:** Spearman correlation coefficients for reaching SCB in ASES, SANE, SST, VAS, and all four outcome measures and outcome metrics of patient overall satisfaction, willingness to undergo surgery again, and willingness to recommend surgery to a friend or family member.

**Reached SCB ASES**	**Spearman Correlation Coefficient**	
Patient Overall Satisfaction	0.014	Very weak
Willing to Undergo Surgery Again	0.035	Very weak
Willing to Recommend Surgery	0.035	Very weak
**Reached SCB SANE**	**Spearman Correlation Coefficient**	
Patient Overall Satisfaction	0.039	Very weak
Willing to Undergo Surgery Again	0.003	Very weak
Willing to Recommend Surgery	0.003	Very weak
**Reached SCB VAS**	**Spearman Correlation Coefficient**	
Patient Overall Satisfaction	−0.011	Very weak
Willing to Undergo Surgery Again	−0.026	Very weak
Willing to Recommend Surgery	0.099	Very weak

**Table 5:** Number of patients that reached PASS in ASES, SANE, and VAS at 2 years postoperatively.

Patient-Reported Outcomes	Number of Patients (N = 92)	
	Reached PASS (%)	Did Not Reach PASS (%)
ASES	56 (60.9%)	36 (39.1%)
SANE	59 (64.1%)	33 (35.9%)
SST	N/A	N/A
VAS	61 (66.3%)	31 (33.7%)

**Table 6:** Spearman correlation coefficients for reaching PASS in ASES, SANE, SST, VAS, and all four outcome measures and outcome metrics of patient overall satisfaction, willingness to undergo surgery again, and willingness to recommend surgery to a friend or family member.

**Reached PASS ASES**	**Spearman Correlation Coefficient**	
Patient Overall Satisfaction	0.073	Very weak
Willing to Undergo Surgery Again	−0.022	Very weak
Willing to Recommend Surgery	0.104	Very weak
**Reached PASS SANE**	**Spearman Correlation Coefficient**	
Patient Overall Satisfaction	0.520	Moderate
Willing to Undergo Surgery Again	0.118	Very weak
Willing to Recommend Surgery	0.118	Very weak
**Reached PASS VAS**	**Spearman Correlation Coefficient**	
Patient Overall Satisfaction	0.166	Very weak
Willing to Undergo Surgery Again	−0.001	Very weak
Willing to Recommend Surgery	0.128	Very weak

## Data Availability

Data is not publically available due to privacy restrictions.
